# Coral cover and species responses to heat exposure vary across contemporary Western Atlantic reefs

**DOI:** 10.1038/s41598-025-28828-3

**Published:** 2025-11-27

**Authors:** Alice E. Webb, Chris T. Perry, F. Javier González-Barrios, John T. Morris, Ruben van Hooidonk, Tyler B. Smith, Donavon R. French, Michelle A. Johnston, Reni Garcia, Maria Vega Rodriguez, Robert R. Ruzicka, Michael A. Colella, Ian C. Enochs

**Affiliations:** 1 Geography, Faculty of Environment, Science and Economy, University of Exeter, Exeter, UK; 2 Lancaster Environment Centre, Lancaster University, Lancaster, UK; 3 Cooperative Institute for Marine and Atmospheric Studies, University of Miami, Miami, Florida USA; 4 Atlantic Oceanographic and Meteorological Laboratory, Ocean Chemistry and Ecosystem Division, National Oceanic and Atmospheric Administration (NOAA), Miami, FL USA; 5 Science for Climate Change Resilience, Boulder, CO 80302 USA; 6 Center for Marine and Environmental Studies, University of the Virgin Islands, Saint Thomas, VI USA; 7 Cardinal Point Captains for Flower Garden, Banks National Marine Sanctuary, Galveston, TX USA; 8 National Oceanic and Atmospheric Administration (NOAA) Office of National Marine Sanctuaries, Flower Garden Banks National Marine Sanctuary, Galveston, TX USA; 9 Reef Research, Inc., P. O. Box 178, Cabo Rojo, PR 00622 USA; 10 PR Department of Natural and Environmental Resources, San Jose Industrial Park, San Juan, PR USA; 11 Fish & Wildlife Research Institute/Florida Fish & Wildlife Conservation Commission, Saint Petersburg, USA

**Keywords:** Climate change, Ocean sciences, Climate-change ecology

## Abstract

Ocean warming threatens the persistence of tropical corals and the biologically diverse ecosystems they sustain. While field-based studies on heat impact have predominantly focused on quantifying coral bleaching, a symptom of thermal stress, less attention has been given to understanding trends in coral mortality, a critical metric for assessing and predicting the long-term effects of rising temperatures. Consequently, the relationships between varying heat exposures and resultant coral cover changes remain poorly quantified. Such trends are challenging to establish in the Tropical and Subtropical Western Atlantic (TWA), because climate change impacts are compounded by local anthropogenic and natural disturbances. Additionally, many coral communities have already been substantially altered, with those remaining dominated by relatively resilient species. This study addresses this issue by quantifying coral cover loss as a function of maximum Degree Heating Weeks (DHW) exposure using observed in-situ coral cover changes across reefs in the TWA. Of the five locations assessed (the Florida Keys, Dry Tortugas, Puerto Rico, the US Virgin Islands, and the East and West Flower Garden Banks), all exhibited significant declines in mean coral cover with increasing DHW exposure. Rates ranged from 0.3% to 2.4% annual loss in relative mean cover per unit DHW, with spatial variability largely reflecting pre-impact system conditions and variations in the life-history traits of geographically distinct coral assemblages (e.g., Puerto Rico vs East and West Flower Garden Banks). Variation in responses to DHW among species was also observed across locations. By establishing site-specific coral loss parameters, this study contributes to our understanding of how future coral cover may evolve under escalating thermal stress in the TWA. It also provides practical guidance for targeted restoration by identifying species that have fared comparatively well across locations, grounding efforts in what is possible under current and future conditions rather than idealised historical baselines.

**Supplementary Information:**

The online version contains supplementary material available at 10.1038/s41598-025-28828-3.

## Introduction

Ocean warming is altering the structure and function of marine communities globally with major impacts on the ecosystem services they provide^1,2^. The frequency and intensity of marine heatwaves has substantially risen due to human-induced greenhouse gas emissions^3,4^. One major consequence in tropical and subtropical waters has been episodic severe coral bleaching, leading to widespread declines in coral cover over the last five decades^5,6^. Coral bleaching occurs when the coral-zooxanthellae symbiosis breaks down. This causes loss of colouration and disruption of essential physiological processes, which can ultimately lead to coral death^7^. Recovery of corals from bleaching is possible but varies greatly between reefs^8–10^, with divergent taxa-level heat stress responses often leading to shifts in coral assemblages^11,12^. Unprecedented high and prolonged heat records over the period 2023-2024 have exposed reefs to new extreme conditions, challenging climate projections and providing critical feedback on coral community persistence under escalating temperatures^13^. Although there has been significant experimental research into the impact of thermal stress on coral physiology and mortality thresholds^14–18^, the relationships between different heat exposures and resultant coral cover changes in the field are not well understood or quantified. To date, most temperature related studies have focused on quantifying coral bleaching, a symptom of thermal stress, rather than coral mortality, a fundamental parameter required to assess the long-term effects of such events (e.g.,^19,20^). Bleaching can lead to decreased coral growth and reproduction, but the capacity of corals to recover means that bleaching incidence does not always accurately predict coral mortality^21^. Establishing these cover response trends to different heat exposure is, however, an essential step towards building predictive models that can guide conservation efforts and inform restoration policy^22,23^.

Metrics of time-integrated sea surface temperature (SST) anomalies, such as Degree Heating Weeks (DHW, ^o^C-weeks), are commonly used to measure accumulated heat stress^24^. Despite the complexities of coral stress responses, DHW stands out as one of the few quantifiable and forecastable indicators of reef stress, providing insights across broad spatial and temporal scales^25,26^. While this metric has limitations, its predictive power remains invaluable, especially as climate models grow increasingly skilled at projecting future thermal stress events^27–29^. DHW exposure has been proven to be an effective tool for the prediction of bleaching in near-real-time (e.g.,^30,31^), however, response curves linking DHW to changes in coral cover are comparatively scarce. The very few studies, to our knowledge, that have identified significant relationships between a range of DHW and associated changes in coral cover are based on global data and mainly focussed on the Indo-Pacific region, where many reefs still retain relatively high coral cover and exhibit notable sensitivity to heat stress^11,32,33^. In contrast, since the rapid loss of corals across the Tropical (and subtropical) Western Atlantic (TWA) that began in the 1970s, coral cover on most reefs has declined to less than 15%^5,34,35^. Over the past two decades, despite continued and increasingly frequent exposure to heat stress events^36^, the rate of coral decline has levelled off^37,38^. This is likely due to several factors, one of which is that many coral communities have already undergone significant alterations and are now dominated by more resilient and resistant species^39,40^. Low coral cover on these reefs, also makes detecting substantial change increasingly difficult^37,41^. Additionally, survey methods like point intercept transects further obscure mortality estimates, as partial mortality from heat stress often goes unrecorded, even when significant damage has occurred. This has complicated the establishment of response trends in this region, especially because the effects of climate change are superimposed on a suite of local anthropogenic and natural disturbances (e.g., coastal development, chronic pollution, high tourism, overexploitation, disease, and hurricanes)^21,42^. How individual reefs will transform under thermal stress can depend on their condition pre-disturbance, including their biogeographic region and site-specific hydrologic conditions, as well as the sensitivity and response capacity to stressors of individual coral species that make up their community^33,43^. This results in a high degree of variability between individual reefs in terms of how they respond to heat^44^, suggesting that relying on global-scale trends for coral cover predictions can obscure the nuanced trajectories of coral decline at specific sites.

Even less common, are studies examining the impact of varying DHW and related changes in cover of different coral taxa. Although the concept of winners versus losers has been widely applied to describe inter-specific differences in the degree of bleaching^45–47^, predicting the definitive losers, namely the species that ultimately die following heat stress, is key to understanding how climate change affects biodiversity, species composition and ecosystem functioning^11^. Establishing local species-specific trends is also crucial for guiding restoration strategies by determining which coral species to outplant and where, while ensuring that targets are aligned with site-specific environmental conditions and the future stressors reefs will face^23^. Given these considerations, there is a need to (re-) evaluate heat-induced cover changes (mean cover and species-specific) on contemporary TWA reefs, especially in the period following mass coral cover decline (~2000 to present), to inform predictive models accordingly^36^. Here, we leverage spatially and temporally extensive datasets from five different locations within the TWA region (the Florida Keys, Dry Tortugas, Puerto Rico, the US Virgin Islands and East and West Flower Garden Banks) to assess the impact of DHW exposure on coral cover of contemporary reefs. The resulting relationships serve as integrative loss parameters reflecting the cumulative effects of thermal stress and all local and historical disturbances at each location. By analysing relative changes in coral cover over a 20-year span, across diverse hydrological regions and reef assemblages, we identify nuanced relationships between DHW and coral cover decline. Drawing on these findings, we then use the established rates of change to forecast coral cover evolution at these locations under two plausible Shared Socioeconomic Pathways (SSP) scenarios (SSP5-8.5 and SSP2-4.5) using site-specific DHW projections.

## Materials and methods

### Data

#### Benthic data

Only data from permanent transects were used for this study to ensure consistent and reliable data collection over time, allowing for accurate tracking of subtle, short-term coral cover changes. This consistency is crucial for establishing relationships between DHW exposure and in-field cover changes. Data analysis was conducted on long-term monitoring datasets from five locations across the northern sector of the TWA biogeographic region, encompassing the Florida Keys, Dry Tortugas, Puerto Rico, US Virgin Islands, and East and West Flower Garden Banks (hereafter East and West FGB). The present analysis incorporates data from 2000 to 2024.

The Coral Reef Evaluation and Monitoring Project (CREMP) in the Florida Keys National Marine Sanctuary has carried out annual assessments since 1996 at fixed sites to evaluate the status and trends of benthic communities. Each CREMP site has four ~22-meter-long survey stations which are permanently marked using steel stakes that were drilled and cemented into the reef. Using a transect tape as a guide, a lightweight plastic chain is laid across the substrate between the two stakes to mark each transect. Present-day CREMP surveys include a photographic survey used to assess percent cover. The data covers 44 reef sites spread across three common habitats (patch reefs n = 15, shallow forereefs n = 12, deep forereefs n = 11, and hardbottom n = 6) in the Florida Keys. Sites range from approximately 1 m to 16 m in depth.

The CREMP also surveys 11 sites annually in the Dry Tortugas. These sites span different coral reef habitats, including unique high-relief pinnacles (n = 5), shallow patch reefs (n = 4), and one spur-and-groove reef (n = 1), that support diverse coral and benthic communities. Approximate depths of sites utilised in this analysis range from 1.5 m to 21 m. Monitoring at some of these locations began in 1999, with additional sites added between 2000 and 2009, encompassing both Dry Tortugas National Park and the Tortugas Northern Ecological Reserve. The data was collected and analysed as in the Florida Keys. All sites have four stations, except for three patch reefs where only three parallel 22 m transects, ~1 m apart, were surveyed.

The Puerto Rico Long-Term Coral Reef Monitoring Program (PRCRMP) database encompasses the Culebra-Vieques islands, the main island of Puerto Rico, Caja de Muertos (south coast of Puerto Rico), and the Desecheo-Mona islands. Coral species cover has been characterised in these locations, with variable sampling event frequencies since 1999. At present, 42 permanent stations (each comprising five 10 m transects) across 18 sites are surveyed every other year (approximately 21 per year). Site depths range from 1.7 m to 20 m.

The US Virgin Islands Territorial Coral Reef Monitoring Program (TCRMP) dataset encompasses the islands of Saint Thomas, Saint John and Saint Croix, in the Northern Caribbean. Since 2001, benthic cover surveys have been conducted annually at each site using six permanent 10 m transects. For this analysis, all mesophotic reefs were excluded, resulting in 23 remaining sites at depths ranging from 6 to 24 m.

The Flower Garden Banks National Marine Sanctuary (FGBNMS) long-term coral reef monitoring dataset captures data from both East FGB and West FGB in the northwest Gulf of Mexico, with surveys spanning the period from 2009 to 2025. These banks are unique ecosystems within the region, in addition to serving as the northernmost coral reefs in the continental United States. Data were collected through annual monitoring efforts that involved repetitive photo stations and stratified random photo transects occurring at depths between 18 and 24 m. At East FGB, 37 photo stations were set within a one-hectare study area divided into four quarters, while West FGB included 41 stations in a similar study area. Mean coral cover used in cover projections for this location is from random transect surveys, as the repetitive photo stations were not representative of the overall community.

#### Satellite-derived DHW data

DHW for all locations were downloaded from the National Oceanic and Atmospheric Administration’s (NOAA) website. Values are calculated using NOA’,s CoralTemp dataset because of its high spatiotemporal resolution and coverage (5km × 5 km daily global values from 1985 to present; NOAA Coral Reef Watch 2018^48^). DHW values represent the accumulation of thermal stress over a 12-week period, combining both the intensity and duration of anomalously warm temperatures. For example, a DHW of four indicates a cumulative heat stress equivalent to four weeks with SSTs 1 °C above the Maximum Monthly Mean (MMM), or two weeks with SSTs 2 °C above the MMM.

### Data treatment

Data was filtered to include only sites shallower than 24 meters. Survey year intervals were restricted to a maximum of three years to allow accurate attribution of peak DHW to changes in coral cover. The relative percent change in coral cover (mean cover and species-specific cover) per year and per individual site was calculated as $\frac{CCf-CCi}{CCi \times t}$, where $CCi$ and $CCf$​ represent initial and final coral cover values, respectively, and $t$ is the time elapsed between measurements. Attributing the most relevant DHW peak to a specific coral cover change depends on the timing of the survey relative to the month of maximum DHW (if a survey took place before the peak in that same year, then that peak should not be assigned to the cover change calculated between that year and the previous one) and to the interval of time between two surveys from which cover change is being calculated.

#### Consecutive years

*Surveys Conducted After Peak DHW of the Year*: If data collection occurs after the DHW peak of the year, the attributed DHW is the maximum peak DHW between the current and previous year as follows:

$$\text{Peak DHW}_{\text{attributed}} \, = \, \text{max} \, \left( {\text{Peak DHW}_{\text{year} - 1} , \, \text{Peak DHW}_{\text{year}} } \right)$$

*Surveys Conducted Before Peak DHW of the Year*: If data collection takes place before the annual DHW peak, then the attributed peak DHW is taken solely from the previous year:

$$\text{Peak DHW}_{\text{attributed}} \, = \, \text{max} \, \left( {\text{Peak DHW}_{\text{year} - 1} } \right)$$

#### Non-consecutive years (one-year gap)

*Surveys Conducted After peak DHW of the Year*: If annual data collection occurs following the year’s DHW peak, the peak DHW value associated with observed cover change is the highest DHW over a three-year window:

$$\text{Peak DHW}_{\text{attributed}} \, = \, \text{max} \, \left( {\text{Peak DHW}_{\text{year}} , \, \text{Peak DHW}_{\text{year} - 1} , \, \text{Peak DHW}_{\text{year} - 2} } \right)$$

*Surveys Conducted Before peak DHW of the Year*: If data collection occurs before the peak DHW of that year, the relative cover change is attributed to the largest DHW peak in the two preceding years:

$$\text{Peak DHW}_{\text{attributed}} \, = \, \text{max} \, \left( {\text{Peak DHW}_{\text{year} - 1} , \, \text{Peak DHW}_{\text{year} - 2} } \right)$$

This approach ensures that the peak DHW value attributed to changes in coral cover represents the maximum thermal stress that could have influenced cover change in the absence of in-year survey data.

For each event where DHW values reached 8 or higher, surveys conducted in the month preceding the peak DHW could also be assigned as the peak DHW for that year. This approach accounted for the ramp-up period leading up to heatwave events (i.e., high DHW already occurring before the peak was reached), as seen during the 2023 and 2024 heatwaves, which recorded maximum DHW values of 18.9 °C-weeks in the Florida Keys, 14.3 °C-weeks in the Dry Tortugas, 11.9 °C-weeks at East and West FGB (2023), and in 2024, 21.3 °C-weeks in Puerto Rico and 21.1 °C-weeks in the U.S. Virgin Islands.

To account for delayed and cumulative effects of heat stress, as well as potential recovery following cooling events, we also derived loss rates using a decay-weighted cumulative DHW index. This index combined peak DHW values from the survey year and the two preceding years, with exponentially decaying weights (half-life = 3 years) and minor timing adjustments for survey dates relative to the annual DHW peak and intervals between survey years. Results of this sensitivity analysis (see Supplementary Materials and Fig S1-S2) were consistent with those from the single-peak DHW metric, supporting retention of the latter in the main analyses for its broader applicability to coral cover projections which are typically based on maxDHW (peak DHW) values rather than weighted DHW indices.

### Data analysis

#### Relative mean coral cover change

To investigate the relationship between annual relative mean coral cover change and DHW at each location, we employed generalised linear mixed models (GLMMs) with a Gaussian error distribution (identity link), including site as a random effect. Although some model assumptions were not perfectly met (in particular for Puerto Rico, East and West FGB and the Florida Keys datasets), and a few outliers were present (as expected given the data’s nature), DHARMa^49^ residual diagnostics indicated no evidence of over- or under-dispersion (dispersion tested non-significant for all models) and showed acceptable residual pattern, supporting the model as a reasonable basis for inference. Additionally, the Gaussian model yielded the lowest AIC relative to alternative distributions and was therefore retained. All analyses were conducted in R^50^ with the *glmmTMB* function from the glmmTMB package^51^. Using the fitted model, predictions were generated across the observed range of DHW values with the *predict_response* function from the ggeffects package in R^52^. This function computes predictions on the response scale, ensuring interpretability in the context of the original data. The predicted values were plotted on top of observed coral cover change. To quantify the strength of the relationship between coral cover change and DHW, the *test_predictions* function from the ggeffects package was applied. This function estimates the slope of the relationship between the dependent variable (cover change) and the predictor (DHW) and provides a 95% confidence interval (CI) for the slope. To illustrate the differences among the effects of max DHW at all the sites, slopes and confidence intervals were plotted. We also evaluated the region-wide impact of DHW using a GLMM in the Gaussian family (identity link), with location as a random intercept and site nested within location to capture site-level variability and between-location differences.

We assessed model generalisation using leave-one-site-out cross-validation (LOSO). Predictive performance was evaluated using RMSE and MAE between predicted and observed values. Despite high point-level variability, the model retained consistent predictive skill across sites (see Supplementary Material and Figure S3 for details).

Because depth can influence thermal exposure, we evaluated its effect by refitting models with depth (m) as an additive term and in interaction with DHW. Depth alone did not improve model fit at any location. A DHW × depth interaction was detected at two locations, indicating a weakening of the DHW effect with depth, but without changing the direction or significance of the DHW term. Given these results, depth was not retained as a core predictor.

#### Absolute mean coral cover change

In addition to the primary focus on relative change, a supplementary analysis of absolute coral cover change was conducted. While absolute change is less transferable across reefs with differing initial states, it provides a direct measure of how cover varies under different DHW conditions. The absolute percent change in coral cover (mean cover) per year and per individual site was calculated as $\frac{CCf-CCi}{ t}$, where CCi and CCf represent initial and final coral cover values, respectively, and t is the time elapsed between measurements (1 or 2 years). All subsequent analyses followed the same procedures as described above for relative cover change and results are available in the Supplementary Materials (Fig S4 and Table S1).

#### Relative species-specific cover change

To examine the relationship between annual species-specific cover change and DHW, we used GLMMs for each species at each location, with a Gaussian error distribution and site included as a random effect. Although the response variable was moderately right skewed, we used a Gaussian error distribution because AIC values were similar to those from Gamma models, sample sizes were large enough to support the Gaussian approximation, and this approach avoided the need to introduce arbitrary constants required for Gamma models, thereby improving interpretability. Some coral cover values were extremely low (ranging between 0 and 0.05%), meaning even small changes could result in disproportionately large relative percentage increases. To mitigate this effect, we excluded cover change values above the 95th percentile for each species. We then applied the same analysis used for mean coral cover to estimate slopes and confidence intervals, which were plotted to examine species-specific responses to thermal stress across sites. This approach facilitated a detailed comparison of how species varied in their sensitivity to DHW at different locations. We then fit the species-level GLMMs using absolute cover change, which required no trimming, to ensure results were consistent in both direction and significance (Table S2). A Benjamini–Hochberg false discovery rate (FDR) correction was applied to control for multiple testing across species-level models for both relative and absolute cover change analyses (Table S2). Finally, to assess the region-wide impact of DHW on each species, we combined data across all locations and fitted a GLMM with a Gaussian distribution, including site nested within location as a random effect to account for site-level variability.

### Coral cover projections

#### Emission scenarios

To predict change in coral cover caused by both the duration and intensity of marine heatwaves on coral reefs, we used the commonly used metric DHW. Monthly SST projections were downloaded from Mellin et al.^53^, specifically from the SSP5-8.5 (high emissions) and SSP2-4.5 (moderate emissions) pathways. SSP5–8.5 is the pathway that represents high continued fossil fuel usage and associated high rates of greenhouse gas emissions. It is considered a “worst case scenario” and assumes either the absence of climate policy or that existing policies are ineffective. SSP2–4.5 is a “middle of the road” pathway that assumes moderate and delayed mitigation efforts, with emissions continuing to rise until around mid-century and then gradually declining due to slowly adopted climate policies and technological advancements. The original MMM values were extracted directly from the DHW files included in the dataset. Degree heating months (DHMs) were then calculated following NOAA Coral Reef Watch protocols, where each monthly SST value exceeding the MMM + 1 °C threshold contributes to thermal stress^54^. Specifically, for each month where SST > MMM + 1 °C, the anomaly (i.e., SST – [MMM + 1 °C]) was accumulated to generate hotspots. These monthly anomalies were added over each 3-month period to calculate DHMs, these were then converted to DHW by multiplying by 4.348 (the average number of weeks per month). This DHW metric is widely used to assess the risk of coral bleaching. All analyses were conducted at the dataset’s original spatial resolution of 0.5° × 0.5°, without spatial interpolation or gap-filling. MaxDHW projections were then used to forecast coral cover loss.

#### Projections

To explore the application of our work, we projected mean coral cover at each location from 2024 to 2100 using maxDHW projections under SSP5–8.5 and SSP2–4.5 scenarios. For each year, projected changes in cover were estimated by multiplying the rate of change (slope coefficient) by the projected maxDHW values. The resulting annual loss was then applied to the previous year’s cover to derive the projected cover for a given year. Projection error was quantified using lower and upper confidence intervals of the estimated DHW effect on coral cover (see above section). All projections assume that corals do not adapt to increasing DHW.

## Results and discussion

### Coral cover loss parameters in the tropical Western Atlantic

**Fig. 1:**
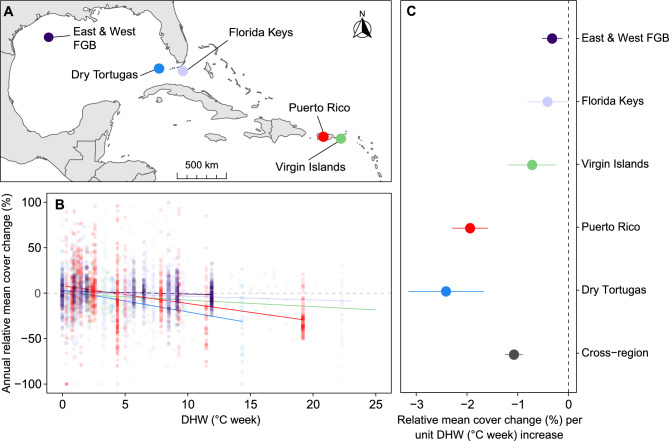
**A** Locations where data were collected. **B** Predicted annual average rate of relative change in mean coral cover (%) as a function of maxDHW for each location. Each point represents the annual relative change in mean coral cover at an individual site within each location. **C** Effect sizes (slopes) from GLMMs, with each point representing the annual rate of relative change in mean coral cover per unit increase in DHW at each location. Error bars indicate 95% confidence intervals and slopes are significantly different from zero if their 95% CIs do not overlap with the vertical dashed line at zero.

In all five locations, relative changes in coral cover in response to increased DHW were significant (Fig 1, Table S3). Among them, the Dry Tortugas and Puerto Rico showed the most pronounced sensitivity, with relative coral cover decreasing by −2.41% (95% CI: −3.15, −1.67) and −1.94% (95% CI: −2.29, −1.59) per unit increase in DHW, respectively. These steeper rates of change align with long-term trends between 2000 and 2024 in these locations where coral cover dropped from 16% to 2.5% in the Dry Tortugas and from 27% to 9.5% in Puerto Rico (Fig S5; see also absolute loss analyses in Fig S4 and Table S1). In the U.S. Virgin Islands, relative coral cover decreased by −0.72% (95% CI: −1.19, −0.24) per unit-DHW increase, with overall cover decreasing from 12% to 7% over the same period. The Florida Keys and East and West FGB exhibited the smallest slopes with −0.41 % (95% CI: −1.08, −0.01) and −0.32 % (95% CI: −0.52, −0.12) change in relative cover per unit-DHW increase respectively, reflecting modest changes over the past ~20 years. Although both these slopes are modest, they reveal very distinct ecological narratives.

In the Florida Keys, the relatively minor effect of DHW on coral cover change likely reflects the already low baseline coral cover in the region (declining from 6.6% in 2000 to 4.5% in 2024, Fig S1), leaving limited remaining corals to be further impacted by thermal stress. The corals that do persist may be either relatively resilient/resistant or exist at such low densities that detecting changes in cover is inherently difficult^37^. This, along with the multitude of other stressors that have impacted the reefs of this region (e.g., disease outbreaks, hurricane damage, poor water quality), has long confounded efforts to isolate the specific effects of thermal stress on coral dynamics in this area. However, the present analysis includes data from the years 2023 and 2024, where heat exposures exceeded 20 DHW on some reefs, leading to the ecological extinction of both *Acropora* species in the Florida Keys and Dry Tortugas^55,88^. These unprecedented events have likely made it possible to detect a significant relationship between DHW and coral cover change.

Of note, the deeper East and West FGB reefs have maintained much higher coral cover levels, which averaged 56% in 2024 (compared to 70% in 2009, Fig S1). This is one of the highest cover levels remaining in the TWA^56^and is likely due, in part, to its remote offshore location and proximity to cooler deeper waters, suggesting that the coral reefs within the sanctuary may be a depth refugium^57^. Further, its designated National Marine Sanctuary status since 1992 has offered additional protections to the benthic habitat such as enforcement of anchoring restrictions, discharge, and bottom-impacting fishing activities. There have been instances of localised coral mortality due to thermal stress-related bleaching^58^, but with relatively rapid recovery leading to limited net long-term changes in coral cover^59^. However, DHW utilised in this study are calculated from satellite-derived SST data which is not representative of site-specific subsurface conditions (where corals are located starting at 18 m). We expect that the smaller rate of change observed is reflective of lower heat exposures at depth and would increase significantly if the surface DHW values reached greater depths. While these conditions provide some protection from direct human impacts, our results indicate that the East and West FGB corals are not immune to the effects of ocean warming and more recently from associated diseases^56^. Although the greater depth of corals at this location may delay the onset of marine heatwave impacts, continued changes in ocean conditions will inevitably raise temperatures at these depths, leading to rapid coral cover loss.

Our calculated coral cover responses to DHW are lower than available data from the Indo-Pacific region^11,32,33^. Hughes et al.^11^, for example, focused on changes across the Great Barrier Reef (GBR) during a bleaching event in 2016 and found that coral cover on average declined by −39, −60, −67, and −90% under 4, 6, 8, and 10 DHW respectively^60^. Work by Condie et al.^61^ presented modelled results suggesting that maximum bleaching mortality reached 100% before 8 DHW exposure among less resilient groups and before 9 to 10 DHW exposure for thermally tolerant corals on the GBR. These rates of coral cover change reflect the higher continued prevalence of more heat-susceptible species in the Indo-Pacific regions and are likely too high for present-day TWA coral species assemblages and genotypes, at least for those in the northern and north-eastern sectors of the region. In comparison, using loss parameters from the current study, 10 DHW exposure in the waters around Dry Tortugas would result in a 24 % relative decrease in mean coral cover. The lower impact of DHW exposure on contemporary TWA coral cover compared to Indo-Pacific coral reefs does not suggest greater resilience of these ecosystems; rather, it reflects the fact that there is far less remaining coral (mean regional cover is <15%^5^) to be affected by further degradation. Additionally, past chronic (e.g.,^62^) and acute (e.g.,^63^) disturbances in many locations have resulted in non-random losses of coral species, and many of these impaired systems are now dominated by opportunistic species^64–66^. Although these species are more likely to resist heat related stressful conditions, they also tend to lock ecosystems into low functional states^2,11,67^.

### Species-level DHW susceptibility

**Fig. 2:**
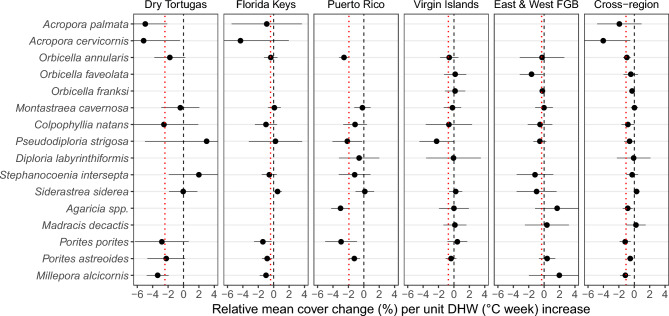
Effect sizes (slopes) estimated from GLMMs, where each point represents the annual rate of relative change in coral cover per unit increase in DHW per species at each location. Error bars indicate 95% confidence intervals (CIs). Positive values reflect an increase, and negative values a decrease, in coral cover with rising DHW. The red dotted lines represent the slope from mean coral cover data (Fig 1c), while the black dashed line marks a slope of zero (no change). The “Cross-region” panel shows GLMM-derived slopes, where each point represents the annual rate of relative coral cover change per unit increase in DHW for each species across all locations. See Supplementary Fig S6 for temporal trends in mean coral cover by taxon at each location. Note: To maintain consistent x-axis scales while allowing visual comparison, some CIs have been truncated.

Quantifying species-level DHW susceptibility identifies which species have contributed to coral cover loss and which have shown more resilience (and/or resistance) over time, while also highlighting variations across and within locations^11^. Although the impact of DHW on coral cover of different taxa is more challenging to interpret than mean cover because of inherent variability and noisiness, some trends have emerged (Fig 2). *Porites astreoides* cover, for instance*,* declined significantly with increasing DHW in Puerto Rico and the Florida Keys, but showed no clear trends elsewhere. Similarly, *Pseudodiploria strigosa* cover declined with DHW in the Virgin Islands and Puerto Rico but appeared relatively stable in other locations*. Porites porites* cover was generally negatively correlated with DHW across most locations, with the exception of the Virgin Islands. *Agaricia* spp. cover was relatively stable at the East and West FGB and in the Virgin Islands but decreased significantly in Puerto Rico. In contrast, *Siderastrea siderea* and *Montastraea cavernosa* cover appeared relatively stable across locations, regardless of DHW. The cross-region panel (Fig 2) illustrates the region-wide impact of DHW on each species. Within the TWA region, *S. siderea* appears to have performed relatively well over the past 20 years, showing an increase in cover with rising DHW. Other species, including *Madracis decactis* and *M. cavernosa*, exhibit relative stability in cover, with little apparent response to changes in DHW. It should be noted that some species, notably brain corals and *Orbicella* spp., are highly susceptible to stony coral tissue loss disease (SCTLD), which may have influenced quantified loss parameters^63^. However, since most sites reported SCTLD only between 2019 and 2022, its impact on most calculated coral cover changes is likely minimal, except in the Florida Keys, where the disease has been present since 2014*.*

These findings highlight the complex and site-specific nature of species’ responses to thermal stress. They may thus help with future efforts to refine restoration targets for these locations, which are hindered by the absence of effective tools to predict how coral cover will evolve under climate change, particularly at the species level^6^. For example, reef restoration initiatives in the Dry Tortugas might benefit from prioritising the outplanting of more tolerant coral species such as *P. strigosa*, *S. intersepta*, and *S. siderea*, rather than relying predominantly on *Acropora* spp. Similarly, in the U.S. Virgin Islands, selecting species such as *M. decactis*, *P porites*., and *S. siderea* may yield more resilient and sustainable restoration outcomes. This approach acknowledges that recovering historical reef assemblages may no longer be achievable and suggests a strategic shift towards restoration practices grounded in ecological feasibility rather than idealised historical baselines.

### Coral cover projections

**Fig. 3:**
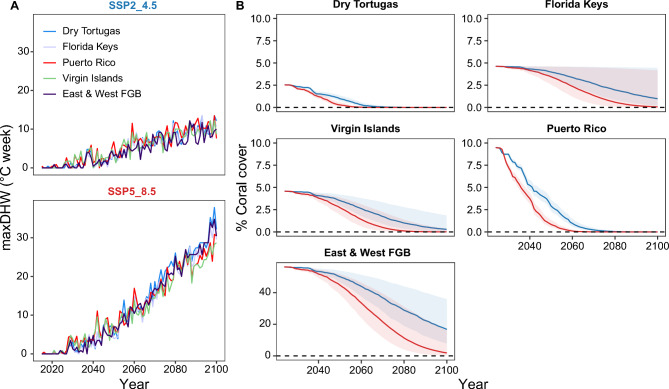
Coral cover projections based on site-specific DHW projections and loss parameters established in this study. **A** MaxDHW projections in each location under SSP2–4.5 (blue) and SSP5–8.5 (red). **B** Mean coral cover projections at each location, under the SSP5–8.5) and SSP2–4.5 scenarios. Error is calculated using lower and upper confidence intervals of the slope to inform coral cover change. Note that the East & West FGB future coral cover is depicted with a different y-axis scale compared to the other locations.

Future trajectories of mean coral cover in each location were projected based on the established relationship between DHW and coral cover change combined with location-specific DHW projections^53^. Initial declines correspond to the timing of rapid increases in DHW (Fig 3). Under the SSP5–8.5 emissions scenario, projections indicate that coral cover in the Dry Tortugas will decline to 0% by approximately 2050, in Puerto Rico by 2060, in the U.S. Virgin Islands by 2080, and in the Florida Keys by 2090. East and West FGB are projected to retain approximately 1% coral cover through to 2100. Emission mitigation under the SSP2–4.5 scenario substantially delays the timeline of coral loss, with the extent of delay varying by location. In the Dry Tortugas and Puerto Rico, local coral extirpation is postponed by roughly a decade, whereas in the U.S. Virgin Islands, the timeline is extended by more than 20 years. In the Florida Keys and East and West FGB, complete coral loss is projected to occur after 2100 under the mitigated scenario. It should be noted that none of these projections apply to deeper mesophotic reef zones. These findings underscore the high spatial variability in coral decline trajectories. While previous projections for the Caribbean have incorporated site-specific DHW estimates (e.g.,^35,60^), they have largely relied on global coral loss parameters. This study contributes refined DHW-coral loss relationships that may be more representative for broader regions of the TWA and further emphasises the value of developing regional or sub-regional models to improve forecasting accuracy.

The underlying assumption of these projections is that coral cover will respond to future temperatures in the same way it does to current temperatures. This is of course unlikely because bleaching thresholds have increased over time due to acclimatisation and adaptation^29,32,68^. This suggests that regardless of the mechanism, projections of future coral based on present-day tolerances may underestimate future adaptive capacity. Additionally, the work by Selmoni et al.^69^ highlights that local factors influence the extent and nature of adaptive responses to changing conditions, indicating that spatial variability in coral responses should also be accounted for in such projections.

### Limitations and considerations

While the DHW metric provides a quantifiable measure of accumulated thermal stress, capturing both the intensity and duration of heat exposure, it only captures the temperature anomaly. Thermal stress has historically played a significant role in reef degradation across the TWA, but it operates alongside multiple, interacting pressures, raising the question of whether DHW can be used as a predictor of coral cover loss^21^. Elevated temperatures also intensify indirect stress pathways, particularly disease^70^. In the TWA, disease has arguably been the primary driver of coral collapse. White-band disease (WBD), strongly linked to high temperatures, decimated *Acropora* populations in the 1980s^71^. While not all coral diseases are strongly linked to temperature (e.g., stony coral tissue loss disease^72,73^), warming seas can still compromise coral immune function and destabilise microbial communities, creating favourable conditions for disease outbreaks. Rising temperatures also exacerbate other stressors, such as nutrient enrichment from coastal runoff, which can trigger algal blooms, hypoxia, and also increase disease susceptibility^74^. Additionally, warming intensifies extreme weather events, which can cause extensive physical damage to reef structures and delay recovery processes^75,76^. To ensure a comprehensive assessment, we included all available observations, rather than restricting the dataset to years with documented bleaching. This approach enabled us to capture not only bleaching-related mortality, but also changes in growth, health, competitive dynamics, disease susceptibility (both increases and reductions), and resilience to subsequent disturbances. This was necessary to evaluate coral cover change across the full DHW gradient, including low-DHW years where little or no bleaching-related mortality occurred. Thus, while DHW serves as a useful metric for quantifying thermal stress, the resulting biological and ecological ramifications extend beyond direct heat exposure, capturing the broader, multifarious impacts of climate change on coral reefs.

The assumption of a linear relationship between DHW and coral cover change may oversimplify the response of reef ecosystems. While in some cases, a higher DHW can correlate with greater coral loss, this is not universally true. Reefs exhibit variable resilience based on factors like species composition, historical exposure to stress, and localised environmental conditions^44,77^. Exposure to repeated thermal stress may also have opposite effects on different reefs. In some cases, consecutive heatwaves may induce acclimatisation, whereby corals exhibit increased tolerance to subsequent events through physiological or symbiotic adjustments^78–82^. Conversely, successive heat events can also lead to sensitisation, particularly if recovery periods are insufficient, resulting in cumulative damage and heightened vulnerability to future stress^80,83,84^. By leveraging extensive datasets that include a wide range of coral cover changes from both standalone heatwaves and repetitive events, our analysis aims to capture these variable impacts. Further, DHW remains one of the few quantifiable and predictable indicators of reef stress over large spatial and temporal scales. Despite its limitations, this predictive capacity is valuable, particularly as climate models become more sophisticated in projecting future thermal stress events^27,28,85^. Altogether, although DHW alone may not capture all the complexities of coral reef dynamics, it provides a valuable baseline for assessing coral cover change in the face of accelerating climate change. By analysing a wide range of coral responses to DHW across different regions and timeframes, we can move closer to establishing tailored loss parameters, guiding more targeted and effective restoration efforts.

Another important consideration of this study is the range of survey depths included in the dataset. Sites span ~1 to 16–24 m (except East and West FGB), and corals are not expected to respond uniformly across this gradient. A significant weakening of the DHW effect with increasing depth was detected only in the Florida Keys (estimate = 0.11 ± 0.05, *p* = 0.027) and the Dry Tortugas (estimate = 0.27 ± 0.06, *p* < 0.001). This may reflect the particularly shallow sites (0.9–3 m) found at both locations compared with others, or that depth-dependent buffering was too limited or variable to detect elsewhere. Because depth was not included in the final models, the slopes represent an average response across the sampled depth range. Nevertheless, by incorporating sites across this depth gradient, the loss parameters established here are broadly applicable to shallow reef habitats within this range.

## Conclusion

The present study identifies for the first time significant relationships between maximum DHW and in-field coral cover change in the TWA. The lack of meaningful and coordinated global action to-date to reduce human impacts, coupled with emerging challenges to address global greenhouse gas emissions, means we are committed to facing rising temperatures along with more frequent and intense heatwaves. Efforts to restore the world’s coral reefs and the likelihood of their success will hinge upon our capacity to predict the trajectory of coral cover in response to climate change, especially at the species level. Coral species exhibit varying levels of resilience across different localities, underscoring the need for tailored, site-specific restoration strategies rather than a global approach^86^. The tailored loss parameters established here can contribute to our understanding of how future coral cover will evolve in this region. This has relevance for predicting changes to key reef functional attributes such as reef framework building^2^, reef capacity to track sea-level rise^87^ and for guiding the design and potential outcomes of targeted restoration efforts. Although essential, local and regional restoration or conservation efforts do not substitute the need for global reductions in carbon emissions. Rather, they should complement broader strategies aimed at ensuring the long-term persistence and recovery of coral reefs.

## Supplementary Information

Supplementary Information 1.

Supplementary Information 2.

Supplementary Information 3.

Supplementary Information 4.

## Data Availability

The data generated in this study are provided in the Supplementary Material accompanying this article. For additional information or enquiries, please contact the corresponding author.
