# Supplementary material for: Coral cover and species responses to heat exposure vary across contemporary Western Atlantic reefs
Source: Sci Rep. 2025 Nov 27;16:45. doi: 10.1038/s41598-025-28828-3 (PMC12764933; doi:10.1038/s41598-025-28828-3)
Supplement: Supplementary file 3 — Supplementary Information 3. [file 41598_2025_28828_MOESM3_ESM.pdf]

Supplementary Materials

Coral cover and species responses to heat exposure vary across contemporary Western Atlantic reefs

Alice E. Webb[1*], Chris T. Perry[1], F. Javier González-Barrios[2], John T. Morris[3,4], Ruben van Hooidonk[5], Tyler B. Smith[6], Donavon R. French[7], Michelle A. Johnston[8], Reni Garcia[9], Maria Vega Rodriguez[10], Robert R. Ruzicka[11], Michael A. Colella[11], Ian C. Enochs[4]

[1]*Geography, Faculty of Environment, Science and Economy, University of Exeter, Exeter, UK*
[2]*Lancaster Environment Centre, Lancaster University, Lancaster, UK*
[3]*Cooperative Institute for Marine and Atmospheric Studies, University of Miami, Miami, Florida, USA*
[4]*Atlantic Oceanographic and Meteorological Laboratory, Ocean Chemistry and Ecosystem Division, NOAA, Miami, Florida, USA*
[5]*Science for Climate Change Resilience, Boulder CO 80302, USA*
[6]*Center for Marine and Environmental Studies, University of the Virgin Islands, Saint Thomas, VI*
[7]*Cardinal Point Captains for Flower Garden Banks National Marine Sanctuary, Galveston, TX, USA*
[8]*National Oceanic and Atmospheric Administration (NOAA) Office of National Marine Sanctuaries, Flower Garden Banks National Marine Sanctuary, Galveston, TX, USA*
[9]*Reef Research, Inc., P. O. Box 178, Cabo Rojo, PR 00622*
[10]*PR Department of Natural and Environmental Resources, San Jose Industrial Park, San Juan, PR, USA*
[11]*Fish & Wildlife Research Institute/Florida Fish & Wildlife Conservation Commission. Saint Petersburg, USA*

**Correspondence and requests for materials should be addressed to A.W. (a.e.webb@exeter.ac.uk).*

Sensitivity analysis

1. Data treatment

Linking a specific change in coral cover to a single DHW peak is complicated by the fact that heat stress can have delayed and cumulative effects, and recent cooling events could also potentially promote recovery. To capture this time-integrated response, we derived loss rates from a decay-weighted cumulative DHW index combining maxDHW in the survey year and the two preceding years.

$$\mathrm{weightedDHW} = \mathrm{w_0 maxDHW_t} + \mathrm{w_1 maxDHW_{t-1}} + \mathrm{w_2 maxDHW_{t-2}}$$

where t is the year of the later survey; $\mathrm{maxDHW_t}$, $\mathrm{maxDHW_{t-1}}$ and $\mathrm{maxDHW_{t-2}}$ are the annual maximum DHW values in the current, previous, and two-years-prior periods; and w0, w1, w2 are weights that sum to 1. By construction, weightedDHW has the same units as DHW.

We use exponentially decaying baseline weights (recent years count more) with 3 years as a half-life to account for substantial carry over effects of heat stress.

Example (half-life H = 3 years). The baseline decay weights are approximately $\mathrm{w_0} = 0.41$, $\mathrm{w_1} = 0.33$, $\mathrm{w_2} = 0.26$. If $\mathrm{maxDHW_t} = 6$, $\mathrm{maxDHW_{t-1}} = 10$ and $\mathrm{maxDHW_{t-2}} = 4$, then

$$\text{weightedDHW} = 0.46 \times 6 + 0.33 \times 10 + 0.26 \times 4 = 6.9 \text{ DHW}$$

To account for surveys being carried out before or after the annual DHW peak and the interval between 2 survey years (1 or 2 years), we apply small, pre-specified timing adjustments to the weights (m_0, $m_1$and m_2):

Surveys in Consecutive Years:

After peak. The baseline decay was applied to keep the ordering, but t and t-1 were emphasised via multipliers $m_0 = 1.40$, $m_1 = 1$, $m_2 = 0.90$.

Before peak. The maxDHW of the current year was down-weighted and shifted influence to prior years ($m_0 = 0.20$, $m_1 = 1.80$, $m_2 = 1.20$), reflecting that most stress relevant to the interval had not yet peaked in year t.

Surveys Two Years Apart:

After peak: The current year remained largest but less dominant than in consecutive-year surveys; $m_0 = 0.20$ and $m_1 = m_2$ (equal weights for t-1 and t-2).

Before peak: Negligible weight was assigned to the current year ($m_0 = 0.05$) and again equal weights for t-1 and t-2 were enforced ($m_1 = m_2$), representing pre-peak sampling with a two-year interval.

For each event where DHW values reached 8 or higher we applied an extreme-event override where surveys conducted in the month preceding the peak DHW could also be assigned a larger weight for that current year. This approach accounted for the ramp-up period leading up to heatwave events (i.e., high DHW already occurring before the peak was reached), as seen during the 2023 and 2024 heatwaves, which recorded maximum DHW values of 18.89 °C-weeks in the Florida Keys, 14.34 °C-weeks in the Dry Tortugas, 11.91 °C-weeks at Flower Garden Banks (2023), and in 2024, 21.25 °C-weeks in Puerto Rico and 21.06 °C-weeks in the U.S. Virgin Islands.

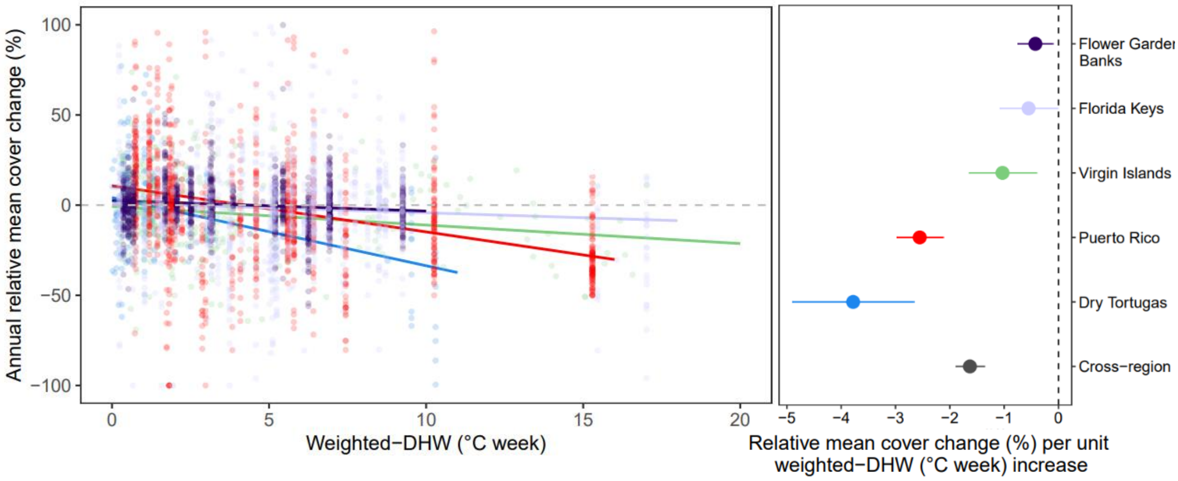

Figure S1. A) Predicted annual rate of relative change in mean coral cover (%) as a function of weighted-DHW for each location. Each point represents the annual relative change in mean coral cover at an individual site within each location. B) Effect sizes (slopes) from generalised mixed linear models (GLMMs), with each point representing the annual rate of relative change in mean coral cover per unit increase in weighted-DHW for each location. Error bars indicate 95% confidence intervals and slopes are significantly different from zero if their 95% CIs do not overlap with the vertical dashed line at zero.

2. Projections

To explore the application of our work, we projected the 2024 mean coral cover at each location. To avoid a scale mismatch, coral cover loss parameters (slopes) were translated from the weighted index scale to the maxDHW scale via a simple linear calibration. For each location, a linear regression was fit between the calculated weighted-DHW and the measured maxDHW, where the slope $a = \mathrm{Cov}(\mathrm{weightedDHW}, \mathrm{maxDHW})/\mathrm{Var}(\mathrm{maxDHW})$ quantifies how much the index slope changes per 1-unit increase in maxDHW. The index-scale slope β (percent relative cover change per unit weighted-DHW) was then converted to a maxDHW-scale slope $\alpha = \beta \times a$.

Projection error was quantified by multiplying the lower and upper confidence-limit estimates of the weighted-DHW slope by a. Coral cover is influenced by multiplying the rate of change (slope coefficient a) by the projected DHW values, assuming corals do not adapt to rising DHW.

Converted rates rates were -2.42% (95% CI: -3.14, -1.70) for the Dry Tortugas, -0.28% (95% CI: -0.54, -0.01) for the Florida Keys, -0.72% (95% CI: -1.16, -0.28) for the Virgin Islands, -1.30% (95% CI: -1.52, -1.08) for Puerto Rico and -0.21% (95% CI: -0.38, -0.05) for the Flower Garden Banks.

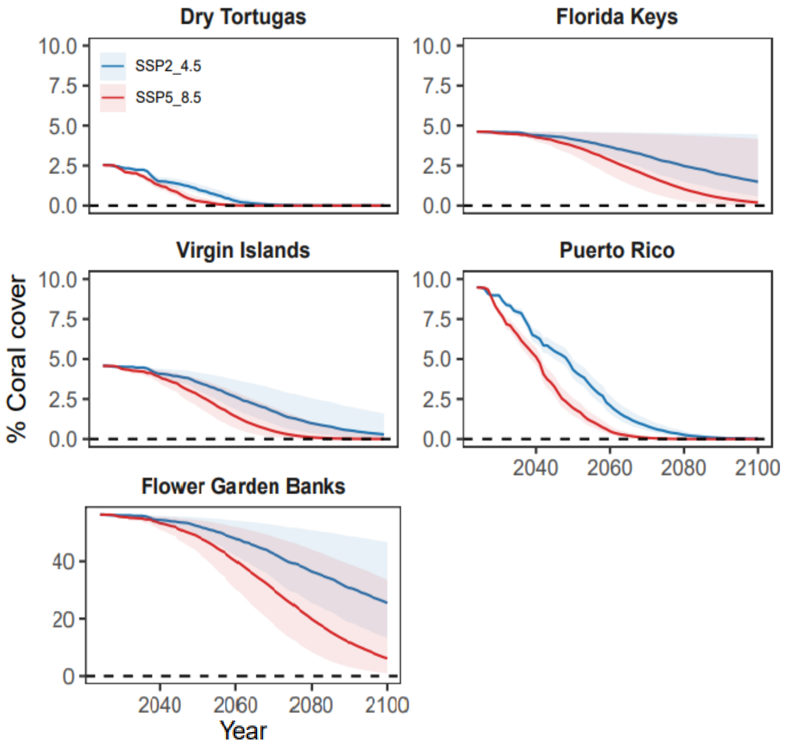

Figure S2. Coral cover projections based on site-specific Degree Heating Weeks (DHW) projections. Loss parameters were established on the weighted-DHW index scale and translated to the maxDHW scale. Mean coral cover projections at each location, under the SSP5–8.5 (red) and SSP2–4.5 (blue) scenarios. Error is calculated using lower and upper confidence intervals of the slope to inform coral cover change. Note that the Flower Garden Banks future coral cover is depicted with a different y-axis scale compared to the other sites.

Leave-one-site-out cross-validation (LOSO)

We assessed model generalisation using leave-one-site-out cross-validation (LOSO), in which models were trained on all but one site and then used to predict the held-out site using only fixed effects. Predictive performance was quantified with root mean squared error (RMSE) and mean absolute error (MAE) between predicted and observed annual relative mean cover change. As expected for field-based ecological data, individual point-level predictions exhibited high variability. To better visualise

model calibration, we aggregated predictions into bins of similar predicted values and compared mean observed outcomes against mean predictions. These binned means (± standard error) closely followed the 1:1 line, indicating that while fine-scale predictions are noisy and point-wise errors (RMSE, MAE) are large due to scatter in the data, the model captures the correct average trend across the prediction range.

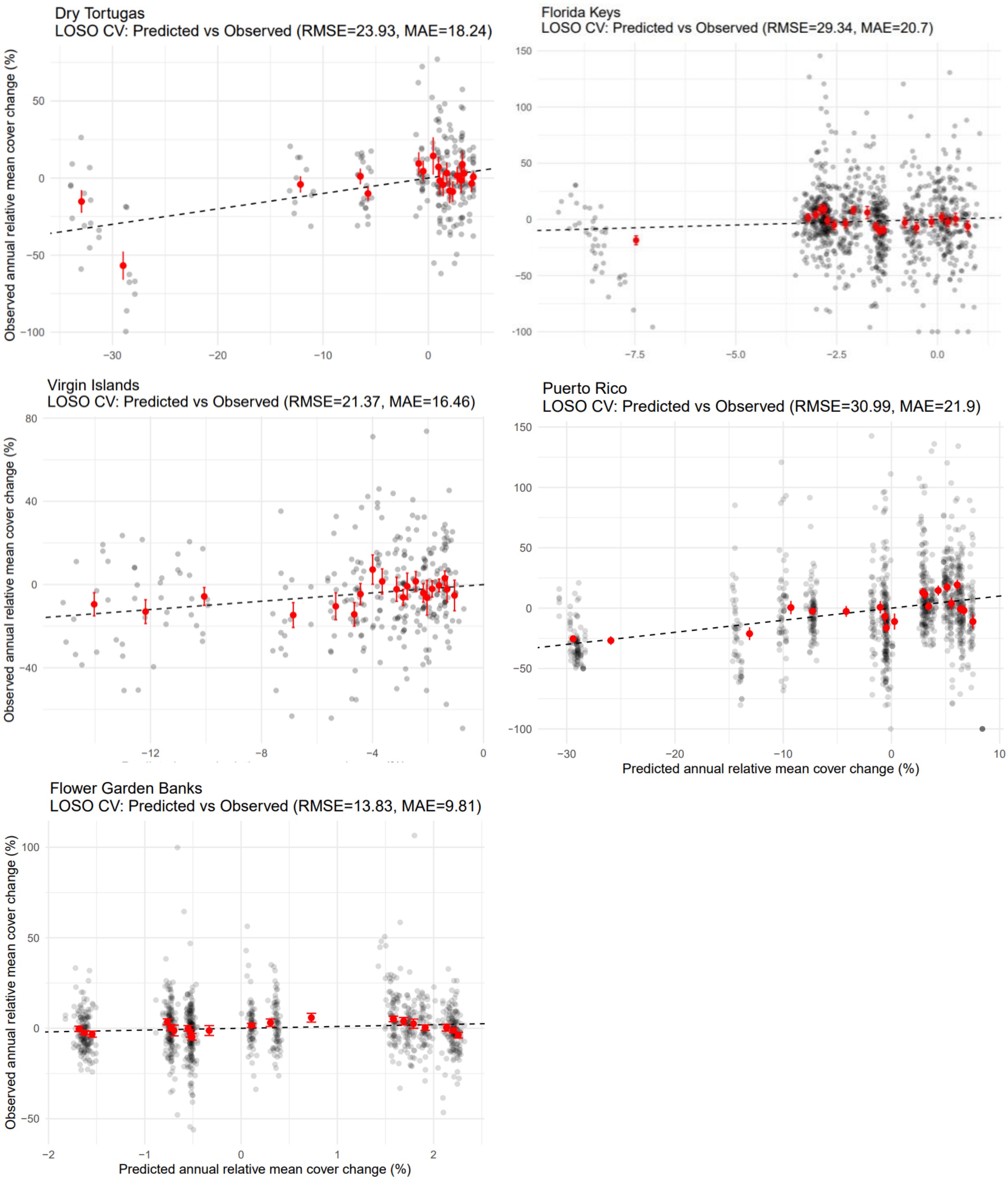

Figure S3. Leave-one-site-out cross-validation (LOSO) predicted vs. observed plot for each location. Grey points = raw predictions, red points ± SE = binned means. Dashed line = 1:1 line.

Absolute cover change

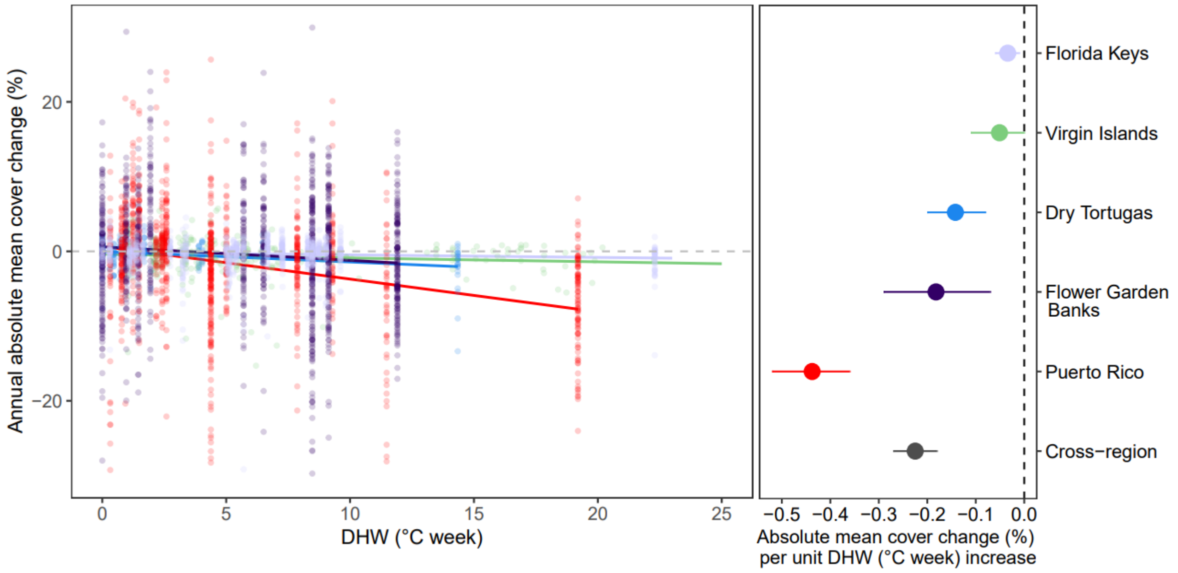

Figure S4. **A)** Predicted annual rate of absolute change in mean coral cover (%) as a function of maxDHW for each location. Each point represents the annual absolute change in mean coral cover at an individual site within each location. **B)** Effect sizes (slopes) from GLMMs, with each point representing the annual rate of absolute change in mean coral cover per unit increase in DHW for each location. Error bars indicate 95% confidence intervals.

Table S1. Table showing the number of species and sites within each location included in the analyses of absolute coral cover change in relation to DHW, along with GLMM-derived p-values and slopes, as well as confidence intervals derived from the test_predictions() function. The number of species included depended on the amount of data available to run the models. All GLMMs incorporated random intercepts for sites to account for spatial non-independence.

**LOCATIONS**	**Florida Keys**	**Dry Tortugas**	**Puerto Rico**	**Virgin Islands**	**Flower Garden Banks**
Sites	46	11	18	23	2
Stations	46	11	42	23	78
Species used in analysis	13	13	14	13	12
Distribution	gaussian	gaussian	gaussian	gaussian	gaussian
Total species	33	30	24	17	23
Slope	-0.03	-0.14	-0.44	-0.05	-0.18
Lower CI	-0.06	-0.2	-0.52	-0.11	-0.29
Upper CI	-0.01	-0.08	-0.36	0	-0.07
SE	0.014	0.029	0.042	0.028	0.058
p	**0.015**	**<0.001**	**<0.001**	0.070	**0.002**
SD Random Int.	<0.001	<0.001	0.2504	<0.001	<0.001

Species-level GLMMS

Table S2. Species-specific loss parameters (slopes) in coral cover change at all five locations. Slopes were estimated using GLMMs with site included as a random effect. For each species, the table reports the slope estimate, 95% confidence interval (CI), unadjusted p value, and false discovery rate–adjusted p value (FDR). Results are presented for both relative cover change and absolute cover change.

FLORIDA KEYS

	Species	Slope	CI Lower	CI Upper	P Value	P Value FDR
**Relative cover change**	*Acropora cervicornis*	-4.309	-10.586	1.968	0.178	0.387
	Acropora palmata	-0.899	-5.463	3.665	0.699	0.827
	Colpophyllia natans	-0.996	-2.434	0.441	0.174	0.387
	Millepora alcicornis	-0.993	-1.811	-0.176	0.017	0.075
	Montastraea cavernosa	0.092	-0.739	0.923	0.828	0.894
	Orbicella annularis	-0.376	-1.250	0.497	0.399	0.576
	Porites astreoides	-0.840	-1.510	-0.170	0.014	0.075
	Porites porites	-1.400	-2.543	-0.257	0.016	0.075
	Pseudodiploria strigosa	0.236	-3.226	3.698	0.894	0.894
	Siderastrea siderea	0.505	-0.053	1.063	0.076	0.247
	Stephanocoenia intersepta	-0.567	-1.546	0.411	0.256	0.456
	Agaricia spp.	-0.422	-1.628	0.784	0.493	0.641
**Absolute cover change**	*Acropora cervicornis*	-0.013	-0.061	0.035	0.603	0.844
	Acropora palmata	-0.010	-0.149	0.129	0.886	0.886
	Colpophyllia natans	-0.020	-0.049	0.009	0.186	0.350
	Millepora alcicornis	-0.013	-0.018	-0.007	0.000	0.000
	Montastraea cavernosa	0.002	-0.009	0.013	0.714	0.844
	Orbicella annularis	-0.032	-0.056	-0.008	0.009	0.044
	Porites astreoides	-0.009	-0.015	-0.002	0.010	0.044
	Porites porites	-0.006	-0.013	0.000	0.054	0.141
	Pseudodiploria strigosa	0.002	-0.018	0.023	0.824	0.886
	Siderastrea siderea	0.002	-0.006	0.009	0.642	0.844
	Stephanocoenia intersepta	-0.004	-0.009	0.002	0.188	0.350
	Agaricia spp.	-0.004	-0.007	0.000	0.054	0.141

DRY TORTUGAS

	Species	Slope	CI Lower	CI Upper	P Value	P Value FDR
**Relative cover change**	*Acropora cervicornis*	-5.179	-9.911	-0.447	0.032	0.091
	Acropora palmata	-4.962	-7.822	-2.103	0.001	0.003
	Colpophyllia natans	-2.541	-6.998	1.916	0.264	0.410
	Millepora alcicornis	-3.355	-4.775	-1.935	<0.001	0.000
	Montastraea cavernosa	-0.413	-2.888	2.062	0.744	0.863
	Orbicella annularis	-1.775	-3.781	0.230	0.083	0.165
	Porites astreoides	-2.243	-4.687	0.200	0.072	0.165
	Porites porites	-2.811	-6.296	0.674	0.114	0.199
	Pseudodiploria clivosa	13.349	1.103	25.596	0.033	0.091
	Pseudodiploria strigosa	2.998	-5.003	10.999	0.463	0.589
	Siderastrea siderea	-0.040	-1.880	1.800	0.966	0.966
	Stephanocoenia intersepta	1.994	-1.910	5.898	0.317	0.443
	Agaricia spp.	-0.602	-5.301	4.096	0.802	0.863
**Absolute cover change**	*Acropora cervicornis*	-0.012	-0.036	0.013	0.352	0.548
	Acropora palmata	-0.362	-0.523	-0.201	0.000	0.000
	Colpophyllia natans	0.005	-0.019	0.029	0.690	0.743
	Millepora alcicornis	-0.029	-0.041	-0.017	0.000	0.000
	Montastraea cavernosa	-0.005	-0.024	0.015	0.646	0.743
	Orbicella annularis	-0.023	-0.050	0.005	0.105	0.183
	Porites astreoides	-0.005	-0.011	0.001	0.085	0.169
	Porites porites	-0.006	-0.013	0.000	0.063	0.147
	Pseudodiploria clivosa	0.013	0.003	0.024	0.014	0.049
	Pseudodiploria strigosa	0.003	-0.005	0.010	0.515	0.721
	Siderastrea siderea	0.002	-0.008	0.013	0.678	0.743
	Stephanocoenia intersepta	0.005	0.000	0.009	0.048	0.135
	Agaricia spp.	0.000	-0.003	0.003	0.946	0.946

PUERTO RICO

	Species	Slope	CI Lower	CI Upper	P Value	P Value FDR
**Relative cover change**	*Agaricia spp.*	-3.024	-4.228	-1.819	<0.001	<0.001
	Colpophyllia natans	-1.138	-2.677	0.401	0.147	0.270
	Diploria labyrinthiformis	-0.610	-3.228	2.008	0.648	0.891
	Montastraea cavernosa	-0.179	-1.235	0.878	0.740	0.904
	Orbicella annularis	-2.572	-3.213	-1.931	<0.001	<0.001
	Porites astreoides	-1.234	-1.979	-0.490	0.001	0.004
	Porites porites	-2.951	-5.017	-0.885	0.005	0.014
	Pseudodiploria strigosa	-2.141	-4.069	-0.212	0.030	0.065
	Siderastrea siderea	0.129	-1.079	1.338	0.834	0.917
	Stephanocoenia intersepta	-1.189	-3.246	0.868	0.257	0.404
**Absolute cover change**	*Agaricia spp.*	-0.038	-0.055	-0.021	0.000	<0.001
	Colpophyllia natans	-0.026	-0.054	0.002	0.068	0.149
	Diploria labyrinthiformis	-0.023	-0.043	-0.003	0.024	0.066
	Montastraea cavernosa	0.010	-0.012	0.032	0.374	0.553
	Orbicella annularis	-0.235	-0.304	-0.167	0.000	<0.001
	Porites astreoides	-0.020	-0.034	-0.006	0.005	0.017
	Porites porites	-0.002	-0.043	0.040	0.936	0.936
	Pseudodiploria strigosa	-0.018	-0.040	0.003	0.098	0.180
	Siderastrea siderea	-0.008	-0.026	0.010	0.402	0.553
	Stephanocoenia intersepta	-0.001	-0.005	0.004	0.831	0.914

VIRGIN ISLANDS

	Species	Slope	CI Lower	CI Upper	P Value	P Value FDR
**Relative cover change**	*Agaricia spp.*	-0.003	-1.940	1.934	0.998	0.998
	Colpophyllia natans	-0.658	-3.667	2.352	0.669	0.998
	Diploria labyrinthiformis	-0.061	-3.607	3.484	0.973	0.998
	Madracis decactis	0.121	-1.368	1.611	0.873	0.998
	Montastraea cavernosa	-0.197	-1.330	0.936	0.733	0.998
	Orbicella annularis	-0.616	-1.802	0.570	0.309	0.998
	Orbicella faveolata	0.159	-1.284	1.601	0.829	0.998
	Orbicella franksi	0.144	-1.167	1.456	0.829	0.998
	Porites astreoides	-0.380	-1.077	0.317	0.286	0.998
	Porites porites	0.437	-0.843	1.716	0.504	0.998
	Pseudodiploria strigosa	-2.256	-4.491	-0.021	0.048	0.670
	Siderastrea siderea	0.228	-0.616	1.073	0.596	0.998
**Absolute cover change**	*Agaricia spp.*	0.009	-0.005	0.023	0.227	0.793
	Colpophyllia natans	-0.033	-0.071	0.005	0.091	0.793
	Diploria labyrinthiformis	0.003	-0.035	0.040	0.894	0.927
	Madracis decactis	-0.004	-0.016	0.008	0.526	0.863
	Montastraea cavernosa	-0.001	-0.019	0.018	0.927	0.927
	Orbicella annularis	-0.023	-0.077	0.032	0.413	0.863
	Orbicella faveolata	-0.012	-0.045	0.021	0.480	0.863
	Orbicella franksi	0.012	-0.048	0.071	0.701	0.863
	Porites astreoides	-0.009	-0.022	0.004	0.181	0.793
	Porites porites	0.006	-0.015	0.026	0.597	0.863
	Pseudodiploria strigosa	-0.030	-0.071	0.012	0.164	0.793
	Siderastrea siderea	-0.002	-0.014	0.010	0.740	0.863

EAST AND WEST FLOWER GARDEN BANKS

	Species	Slope	CI Lower	CI Upper	P Value	P Value FDR
**Relative cover change**	*Agaricia spp.*	1.69	-1.168	4.548	0.247	0.612
	Colpophyllia natans	-0.53	-2.125	1.066	0.515	0.67
	Madracis decactis	0.362	-2.494	3.218	0.804	0.925
	Millepora alcicornis	1.973	-1.97	5.916	0.327	0.612
	Montastraea cavernosa	-0.017	-1.16	1.126	0.977	0.977
	Orbicella annularis	-0.272	-3.165	2.621	0.854	0.925
	Orbicella faveolata	-1.637	-3.157	-0.117	0.035	0.341
	Orbicella franksi	-0.23	-0.619	0.159	0.247	0.612
	Porites astreoides	0.392	-0.662	1.445	0.466	0.67
	Pseudodiploria strigosa	-0.553	-1.396	0.29	0.198	0.612
	Siderastrea siderea	-0.978	-3.568	1.611	0.459	0.67
	Stephanocoenia intersepta	-1.183	-3.559	1.194	0.329	0.612
**Absolute cover change**	*Agaricia spp.*	0.028	-0.038	0.094	0.404	0.813
	Colpophyllia natans	-0.027	-0.103	0.05	0.496	0.813
	Madracis decactis	-0.019	-0.077	0.039	0.515	0.813
	Millepora alcicornis	0.034	-0.068	0.137	0.512	0.813
	Montastraea cavernosa	0.016	-0.07	0.102	0.721	0.937
	Orbicella annularis	-0.329	-0.772	0.115	0.147	0.636
	Orbicella faveolata	-0.157	-0.326	0.012	0.069	0.446
	Orbicella franksi	-0.191	-0.321	-0.061	0.004	0.053
	Porites astreoides	0.002	-0.049	0.053	0.949	0.996
	Pseudodiploria strigosa	-0.004	-0.084	0.076	0.922	0.996
	Siderastrea siderea	-0.176	-0.549	0.198	0.357	0.813
	Stephanocoenia intersepta	-0.021	-0.091	0.05	0.563	0.813

Supporting Tables and Figures

Table S3. Table showing the number of species and sites within each location included in the analyses, along with p-values and slopes from the GLMMs, as well as confidence intervals derived from the test_predictions() function. The number of species included depended on the amount of data available to run the models. All GLMMs incorporated random intercepts for sites.

**LOCATIONS**	**Florida Keys**	**Dry Tortugas**	**Puerto Rico**	**Virgin Islands**	**Flower Garden Banks**
Sites	46	11	18	23	2
Stations	46	11	42	23	78
Species used in analysis	13	13	14	13	12
Distribution	gaussian	gaussian	gaussian	gaussian	gaussian
Total species	33	30	24	17	23
Slope	-0.41	-2.41	-1.94	-0.72	-0.32
Lower CI	-0.81	-3.15	-2.29	-1.19	-0.52
Upper CI	-0.01	-1.67	-1.59	-0.24	-0.12
SE	0.204	0.377	0.177	0.244	0.102
p	**0.043**	**<0.001**	**<0.001**	**0.003**	**0.002**
SD Random Int.	0.001	<0.001	2.955	<0.001	<0.001

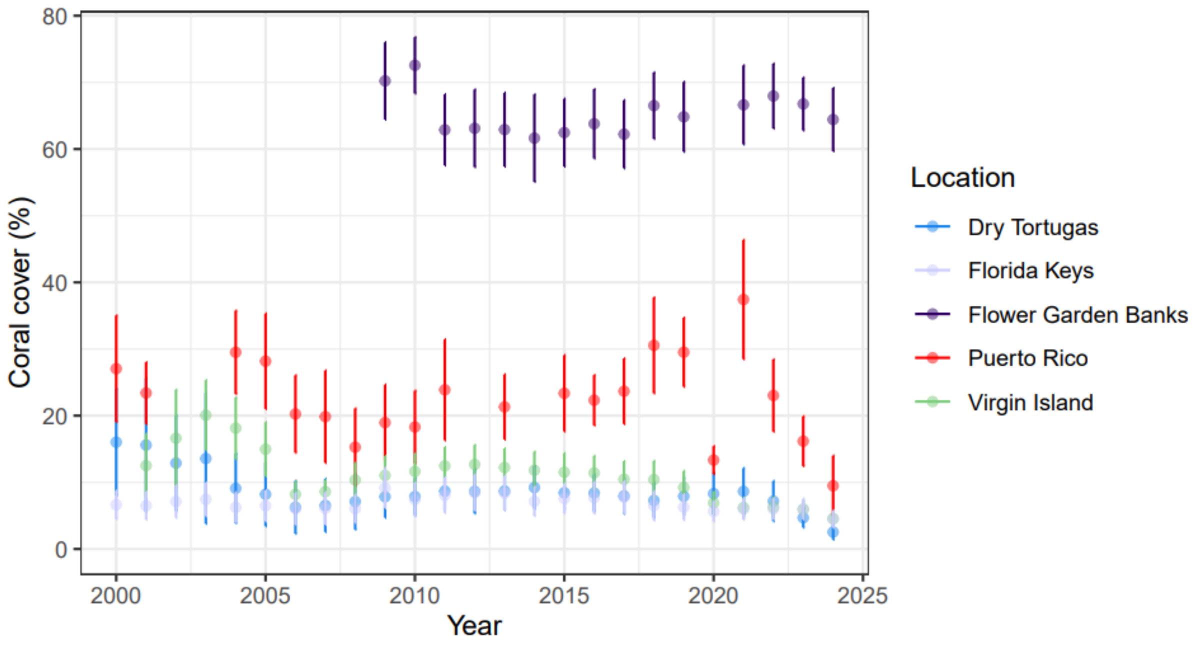

Figure S5. Mean total coral cover (%) and 95% confidence intervals over time at each location.

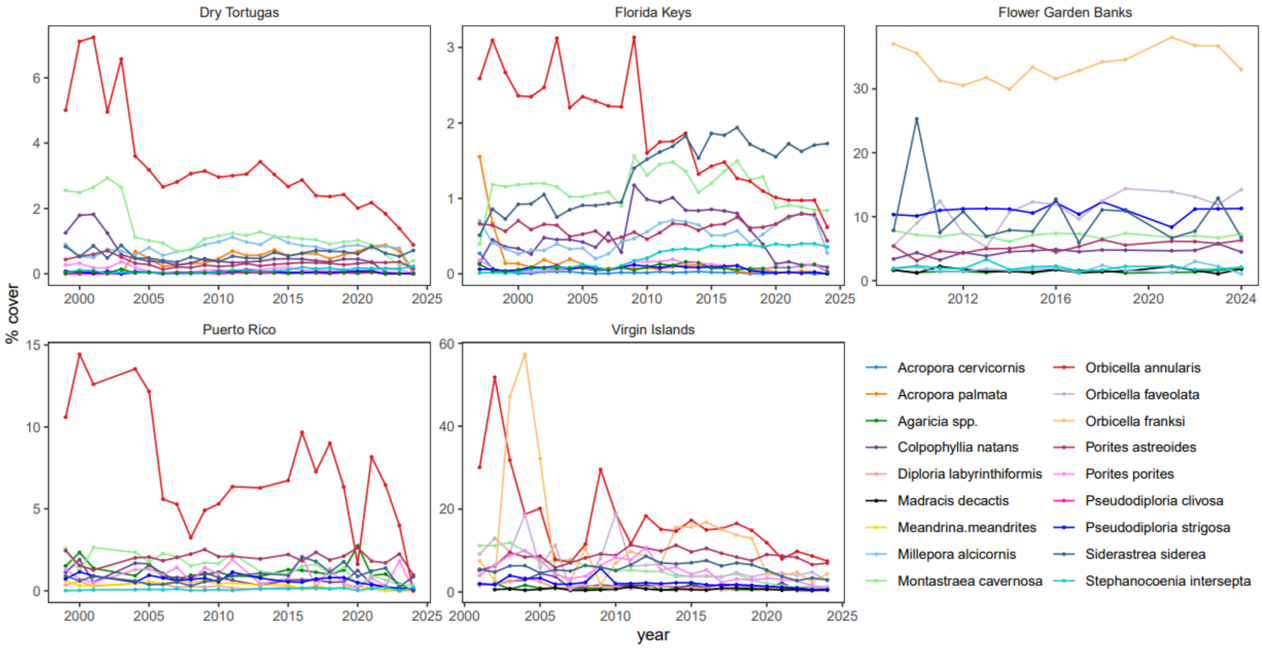

Figure S6. Percent cover of main 18 species found in study locations.
